# Galectin-8 Promotes Cytoskeletal Rearrangement in Trabecular Meshwork Cells through Activation of Rho Signaling

**DOI:** 10.1371/journal.pone.0044400

**Published:** 2012-09-04

**Authors:** Shiri Diskin, Wei-Sheng Chen, Zhiyi Cao, Smita Gyawali, Haiyan Gong, Andrea Soza, Alfonso González, Noorjahan Panjwani

**Affiliations:** 1 Program in Cell, Molecular & Developmental Biology, Sackler School of Graduate Biomedical Sciences, Tufts University, Boston, Massachusetts, United States of America; 2 New England Eye Center and Department of Ophthalmology, Tufts University, Boston, Massachusetts, United States of America; 3 Department of Ophthalmology, Boston University School of Medicine, Boston, Massachusetts, United States of America; 4 Departamento de Inmunología Clínica y Reumatología, Facultad de Medicina, Centro de Regulación Celular y Patología and Centro de Envejecimiento y Regeneración, Facultad de Ciencias Biológicas, Pontificia Universidad Católica de Chile, Santiago, Chile; 5 Millennium Institute for Fundamental and Applied Biology, Santiago, Chile; 6 Department of Biochemistry, Tufts University, Boston, Massachusetts, United States of America; Schepens Eye Research Institute, Harvard Medical School, United States of America

## Abstract

**Purpose:**

The trabecular meshwork (TM) cell-matrix interactions and factors that influence Rho signaling in TM cells are thought to play a pivotal role in the regulation of aqueous outflow. The current study was designed to evaluate the role of a carbohydrate-binding protein, galectin-8 (Gal8), in TM cell adhesion and Rho signaling.

**Methods:**

Normal human TM cells were assayed for Gal8 expression by immunohistochemistry and Western blot analysis. To assess the role of Gal8 in TM cell adhesion and Rho signaling, the cell adhesion and spreading assays were performed on Gal8-coated culture plates in the presence and the absence of anti-β_1_ integrin antibody and Rho and Rho-kinase inhibitors. In addition, the effect of Gal8-mediated cell-matrix interactions on TM cell cytoskeleton arrangement and myosin light chain 2 (MLC2) phosphorylation was examined.

**Principal Findings:**

We demonstrate here that Gal8 is expressed in the TM and a function-blocking anti-β_1_ integrin antibody inhibits the adhesion and spreading of TM cells to Gal8-coated wells. Cell spreading on Gal8 substratum was associated with the accumulation of phosphorylated myosin light chain and the formation of stress fibers that was inhibited by the Rho inhibitor, C3 transferase, as well as by the Rho-kinase inhibitor, Y27632.

**Conclusions/Significance:**

The above findings present a novel function for Gal8 in activating Rho signaling in TM cells. This function may allow Gal8 to participate in the regulation of aqueous outflow.

## Introduction

Primary Open Angle Glaucoma (POAG) is a major cause for irreversible blindness. Factors that lead to the development of POAG are not yet fully known. It is clear, however, that elevated intraocular pressure is a major causal risk factor [1]. Elevation in intraocular pressure is due to the dysfunction of outflow pathway tissues resulting in inadequate clearance of aqueous humor. Trabecular meshwork (TM) cell-matrix adhesion is crucial for the maintenance of the outflow pathway. In the short term, experimental procedures that cause loss of TM cell contact with the beams lead to a sharp increase in aqueous outflow [2], [3], [4]. In the long term, beams denuded of cells (typical of POAG eyes) tend to collapse on one another, blocking the outflow channels [5]. Also, off-target effects of glucocorticoid decrease outflow facility by increasing cell rigidity in TM cells [6], [7], [8]. In recent years, a large body of research in the field of POAG has focused on the role of Rho signaling in the regulation of outflow facility through regulation of the TM actin cytoskeleton. The emerging paradigm is that the inhibition of Rho signaling leads to elevation in outflow facility, while induction of Rho signaling leads to increased resistance to outflow [2], [9], [10], [11]. In cultured TM cells, inhibitors of the Rho signaling cascade cause TM cell rounding, loss of stress fibers and focal adhesions and retraction of cell processes [9], [12]. In perfused human and animal eyes, inhibitors of Rho signaling cause TM cell rounding and detachment from the beams concomitant with a marked elevation in outflow facility [2], [4]. A recent phase 2b clinical trial that utilizes a novel and potent Rho kinase inhibitor, AR-12286, further strengthens the therapeutic importance of relaxing the TM by targeting Rho signaling [13].

In non-ocular studies, a carbohydrate-binding protein, galectin-8 (Gal8), has been shown to form high-affinity interactions with integrins, modulate cell-matrix interactions, and promote cell spreading by activating PI3K and the small GTPases, Ras and Rac [14], [15], [16]. Little is known about the role of the carbohydrate-mediated recognition systems in TM cell adhesion and signaling. In a recent study, we have observed that TM cells adhere to Gal8 substratum and that β_1_ integrins derived from TM cells bind to Gal8 in a carbohydrate-dependent fashion [17]. The role of Gal8 in the regulation of Rho signaling that modulates stress fiber formation and focal adhesion assembly has, thus far, not been investigated in any cell type. In the current study, we underscore for the first time the function of Gal8 in modulating the Rho signaling pathway in TM cells. We demonstrate here that: β1 integrin function-blocking antibody inhibits the adhesion and spreading of TM cells on Gal8-coated wells; cells adhered to Gal8 accumulate phosphorylated myosin light chain 2 (MLC2), and accumulation of phosphorylated MLC2 is associated with stress fiber formation that is abolished by the presence of either the Rho inhibitor, C3 transferase, or the Rho-kinase (ROCK) inhibitor, Y27632. These data lead us to propose that Gal8 promotes cytoskeletal rearrangement in TM cells through interaction with β_1_ integrins leading to activation of the Rho/ROCK/MLC2 signaling pathway.

## Results

### Galectin-8 is Expressed in Human TM Tissue and in Cultured TM Cells

**Figure 1:**
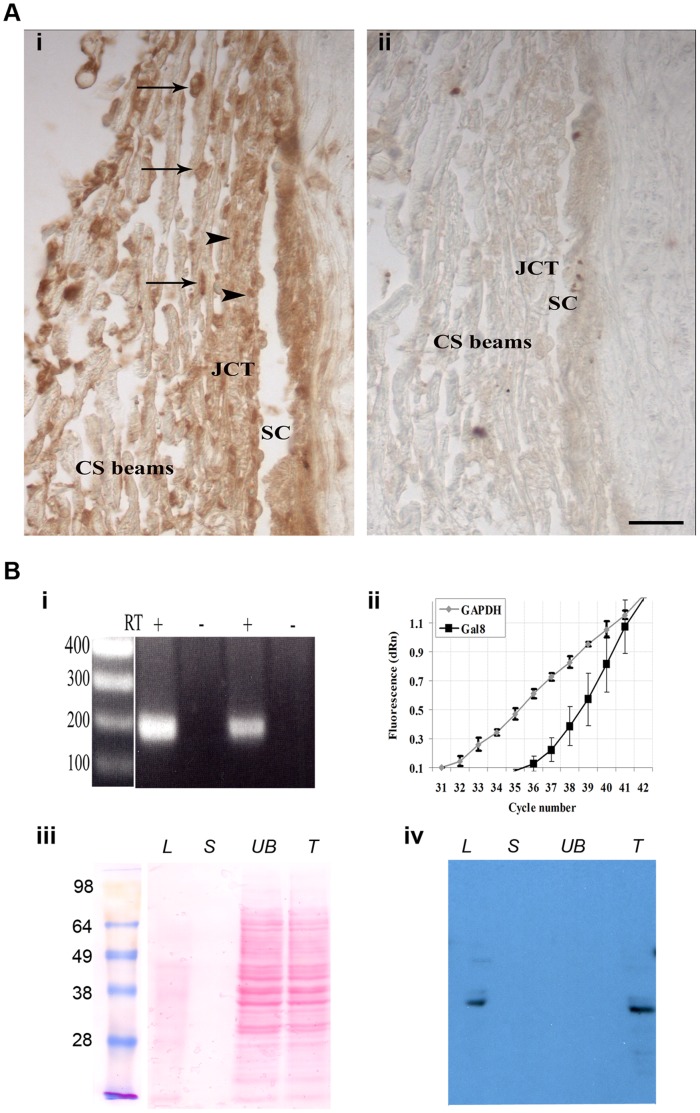
Galectin-8 is expressed in the Trabecular Meshwork (TM). **A:** paraffin sections of anterior chamber angle from a normal human eye were immunostained with anti-Gal8 antibody. (i) anti-Gal8 IgG reacted intensely with cells on the trabecular beams (arrows) and with cells in the juxtacanalicular portion of TM (arrowheads). Staining was also observed in the ECM of both portions of TM (JCT and CS) and in the wall of Schlemm’s canal. (ii) No staining was observed when the sections were not exposed to the primary antibody. *SC*: Schlemm’s canal, *JCT*: juxtacanalicular TM; *CS beams*: corneoscleral beams. Bar: 25 µm. **B:** (i) *RT-PCR*. Total RNA (1.0 µg) from confluent cultures of normal human TM cells was subjected to RT-PCR. The expected 191 bp fragment was amplified using Gal8- specific-primers. In each case, no components were amplified when reaction mixtures lacked reverse transcriptase (RT). (ii) *qRT-PCR*. Total RNA was subjected to Taq-Man RT-PCR using Gal8 specific primers. Original amplification plots of Gal8 and GAPDH mRNAs genes are shown (Ct 37.37 and 33.38 for Gal8 and GAPDH, respectively). N = 3 for each experiment; all experiments were performed twice using TM cells from two different donors with reproducible results. (iii and iv) *Western Blot Analysis*. Protein extracts from confluent cultures of normal human TM cells were incubated with lactogel beads and eluted first with sucrose, and then with lactose. Eluted proteins were electrophoresed, the protein blot of the gel was stained with Ponceau S (iii) and was then processed for immunostaining with goat anti-Gal8 (iv). Both the total cell extract (*T*) and the lactose eluate (*L*) contained a major 36-kDa anti-Gal8 reactive component. This component was not detected in the unbound fraction (*UB*) and in the sucrose eluate (*S*).

Multiple techniques including RT-PCR, Western blot and immunohistochemical staining were used to detect Gal8 expression in TM. In immunohistochemical staining, anti-Gal8 reacted strongly with the TM cells on beams (Figure 1Ai, arrows) and with TM cells in the juxtacanalicular region (Figure 1Ai, arrowheads). Some staining was observed in the ECM in all parts of the tissue and in the wall of Schlemm’s canal (Figure 1Ai, SC). No staining was observed when sections were not exposed to a primary antibody (Figure 1Aii) or exposed to nonimmune goat IgG (data not shown). To assess the expression of Gal8 in human cultured TM cells, RT-PCR experiments were performed on total RNA preparations from two different donors. All RNA preparations produced the expected size Gal8 (191 bp) product (Figure 1Bi). In all cases, when reaction mixtures lacked RT, no components were amplified (Figure 1Bi). Bands were isolated from the gel and sequenced. The product was found by BLAST analysis to be 98% identical to the published Gal8 cDNA sequence [16]. To assess the abundance of Gal8 expression in TM, the expression level of Gal8 was compared with that of GAPDH by quantitative RT-PCR. The qPCR experiments were performed in triplicates on whole RNA extracts from cultured TM cells derived from two different donors. In each case, a robust Gal8 signal was detected (Ct: 37.37 and 33.38 for Gal8 and GAPDH, respectively) (Figure 1Bii). To verify Gal8 protein expression, TM cell lysates were incubated with β-lactose conjugated beads, proteins bound to the beads were eluted first with sucrose, a non-competing sugar, and then with β-lactose, a competing sugar. Both the lactose eluate (Figure 1Biv, lane *L*) and the unfractionated total extract (Figure 1Biv, lane *T*) contained a major anti-Gal8-reactive 36-kDa band, which is the published molecular weight for human Gal8 [16]. This band was absent from the sucrose eluate as well as from the unbound fraction (Figure 1Biv, lanes *S* and *UB*). Many other protein bands in both the total extract and unbound fractions that were detected by Ponceau S staining (Figure 1Biii, lanes *UB* and *T*) were not stained with anti-Gal8, thereby attesting to the high specificity of the antibody. No bands were visible in control immunoblots that were not exposed to primary antibody (data not shown).

### TM Cell Adhesion and Spreading on Gal8 is Mediated by β_1_ Integrins

**Figure 2:**
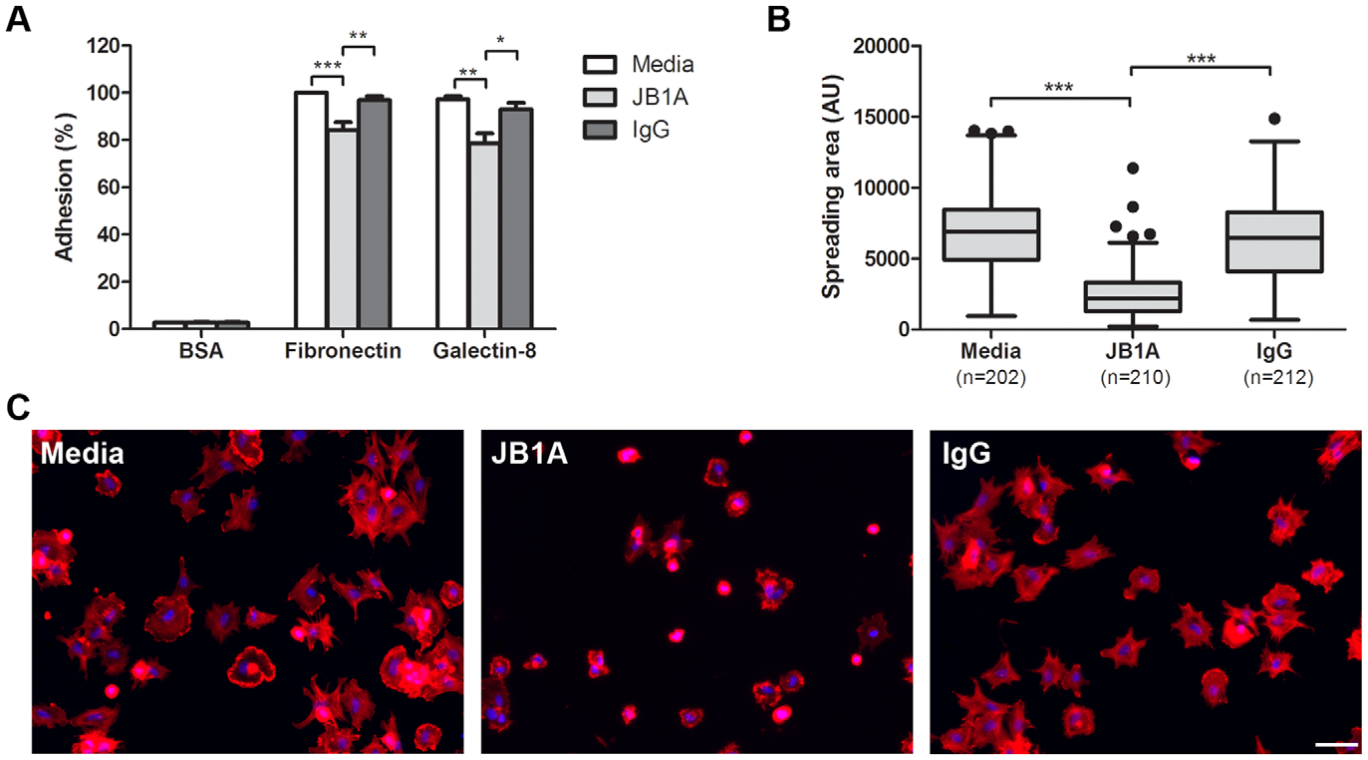
TM cell adhesion and spreading on Gal8 is mediated by β1 integrins. A: Normal human TM cells were incubated on microtiter wells coated with BSA, fibronectin, or Gal8 in DPBS, in the presence and absence of a function-blocking anti-β_1_ integrin antibody (JB1A), or control mouse IgG. Following incubation at 37°C for 30 min, cells were fixed and stained with crystal violet. Attached cells in fibronectin-coated wells are set as 100% (positive control); attached cells in other wells are presented as percent of positive control. Data are expressed as mean±SEM and analyzed with one-way ANOVA. *P<0.05 vs IgG; **P<0.01 vs media or IgG; ***P<0.001 vs media. **B** and **C: Cell spreading assay.** TM cells were fixed with 4% paraformaldehyde after adhesion for 30 min. F-actin was stained with rhodamine-labeled phalloidin and cell nuclei were labeled with DAPI. Random fields of each experimental condition were photographed, and spread areas of individual cells were quantified with ImageJ. Representative micrographs of TM cells incubated in the presence and the absence of anti-β_1_ integrin antibody are shown in C. Data are presented as Box–whisker plot (after Tukey) and analyzed with one-way ANOVA. ***P<0.001 vs media or IgG. This experiment was performed three times with reproducible results. Bar: 100 µm.

In a recent study, we observed that TM cells adhere to and spread on Gal8 substratum and that β_1_ integrins derived from TM cells bind to Gal8 in a carbohydrate-dependent fashion [17]. Because β_1_ integrins are known to play a central role in cell adhesion and spreading [18], [19], [20], we sought to determine whether the adhesion and spreading of TM cells to Gal8 is mediated by β_1_ integrins. For this, we conducted the cell adhesion assay in the presence and the absence of a function-blocking anti-β_1_ integrin antibody (JB1A). Anti-β_1_ integrin antibody inhibited cell adhesion to both Gal8 and fibronectin (positive control) by about 20% (Figure 2A). The extent of inhibition of cell adhesion to fibronectin by anti-β_1_ integrin observed in our study is substantially lower than the published values for a number of nonocular cell types [21], [22], [23], but is consistent with a published study reporting that anti-β_1_ integrin antibodies attenuate TM cell adhesion to fibronectin by 10–20% [20]. F-actin staining revealed that anti-β_1_ integrin antibody abolished cell spreading on Gal8-coated wells, whereas control IgG had no such effect (Figures 2B and 2C). Taken together, these data suggest that TM cell adhesion and, in particular spreading on Gal8, is mediated by one or more β_1_ integrins.

### Galectin-8 Promotes Rearrangement of Cytoskeleton and Modulates Rho Signaling in TM Cells

**Figure 3:**
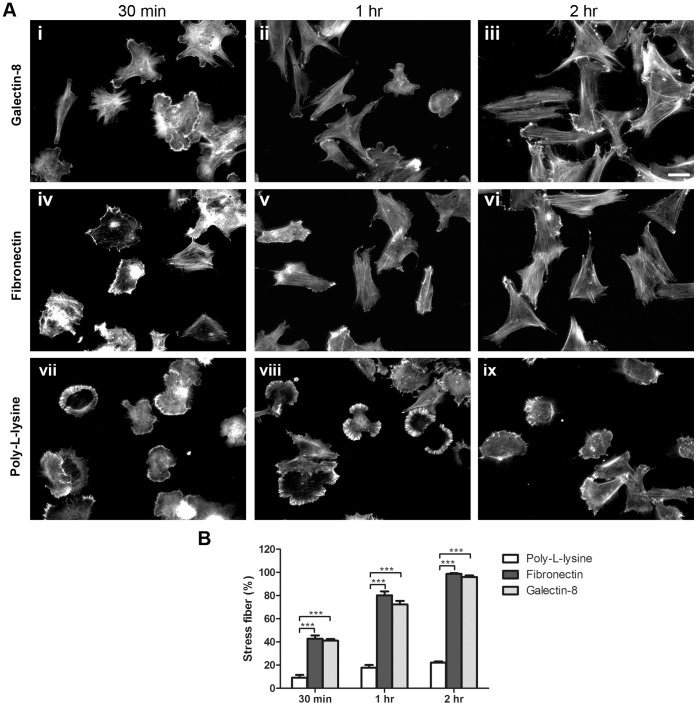
Galectin-8 promotes cytoskeletal rearrangement in TM cells. **A:** Normal human TM cells were plated on eight-chamber glass slides coated with 20 µg/ml of recombinant human Gal8 (i–iii), 20 µg/ml of fibronectin (iv–vi), or 100 µg/ml of poly-L-lysine (vii–ix) in serum-free DMEM at 37°C for 30 min (i, iv, vii), 1 hr (ii, v, viii), and 2 hr (iii, vi, ix). Following the incubation period, cells were fixed with 4% paraformaldehyde and stained with rhodamine-labeled phalloidin. Bar: 50 µm. **B:** Quantification of stress fiber formation. Random fields were photographed, and cells with robust stress fibers were counted. N = 225 to 362. Data are expressed as mean±SEM and analyzed with one-way ANOVA. ***P<0.001 vs poly-L-lysine at different time points. This experiment was performed three times with reproducible results.

**Figure 4:**
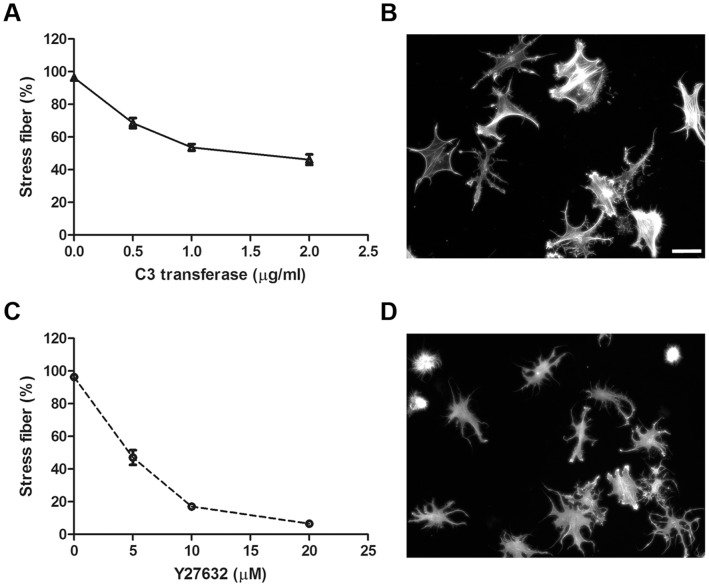
Rho and ROCK inhibitors inhibit Galectin-8 induced stress fiber formation. **A** and **C:** Serum-starved human TM cells were incubated on chamber glass slides coated with recombinant human Gal8 in the presence of the Rho inhibitor, C3 transferase or ROCK inhibitor, Y27632, at different concentrations. After 2 hr, cells were stained with rhodamine-labeled phalloidin, and cells with robust stress fibers were enumerated. Data are expressed as mean±SEM. **B** and **D:** Cells were treated C3 transferase at 2 µg/ml (B) or Y27632 at 20 µM (D), stained with rhodamine-labeled phalloidin, and random fields were photographed. Note that cells treated with Y27632 or C3 transferase are not spread and exhibit dendrite-like structures. Bar: 50 µm.

Having established that Gal8 modulates TM cell spreading, it was of interest to determine whether the lectin has the capacity to influence the structure of cytoskeleton and Rho signaling. To determine whether Gal8 promotes rearrangement of the actin cytoskeleton, TM cells incubated on slides coated with Gal8 for varying periods (30 min to 2 hr) were stained with rhodamine-labeled phalloidin. Slides coated with human fibronectin and poly-L-lysine served as positive and negative controls, respectively. Forces generated by actin polymerization drive cells to form membrane protrusion. Actin punctate staining indicates the initiation of actin polymerization. Cells incubated on Gal8 for 30 min showed membrane protrusions and the actin cytoskeleton showed punctate staining (Figure 3Ai). Following 1 hr incubation, initial stress fibers were seen (Figure 3Aii), and at 2 hr, the cells were fully spread and contained stress fibers aligned along the longitudinal axes of the cells (Figure 3Aiii). A very similar transition from rounded cells with punctate actin staining to spread cells with stress fibers was observed in cells adhered to fibronectin (Figure 3Aiv–vi). Cells with robust stress fibers were quantified at different time points. After 2 hr incubation, more than 95% of TM cells formed stress fibers on Gal-8 as well as fibronectin substrate (Figure 3B). In contrast, most cells adhered to poly-L-lysine retained their rounded shape and punctate actin staining up to 2 hr incubation in serum-free medium (Figure 3Avii–ix). These results suggest that the interaction between integrins and Gal8 which leads to cell spreading is indeed mediated through rearrangement of the actin cytoskeleton and formation of stress fibers. To determine whether stress fiber formation in TM cells adhered to Gal8 is mediated by Rho GTPase; we conducted the cytoskeletal rearrangement assay in the presence of a Rho specific inhibitor, C3 transferase. Cells incubated on Gal8 for 2 hr, showed fully developed stress fibers (Figure 3Aiii). When cells were incubated on Gal8-coated glass slides in the presence of C3 transferase, the formation of actin stress fibers was significantly reduced in a dose-dependent manner (Figure 4A). Cells were spread, but only a few isolated stress fibers formed, and actin staining became predominantly punctate. In addition, some cells were characterized by collapse of the cell body and protrusion of dendrite-like extensions (Figure 4B).

**Figure 5:**
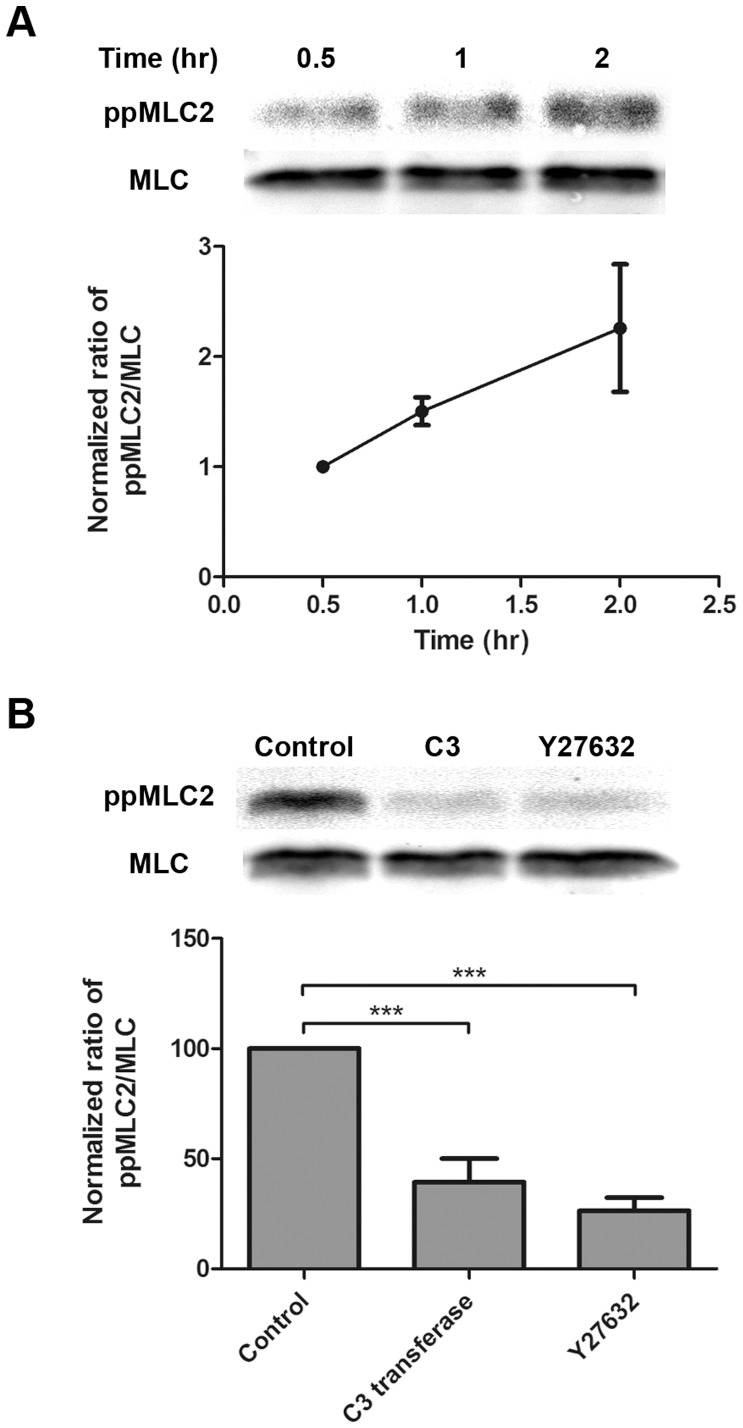
Galectin-8 promotes phosphorylation of myosin light chain 2 in a Rho- and ROCK- dependent manner. **A:** Gal8 induces phosphorylation of MLC2 in a time-dependent manner. Normal human TM cells were incubated on 100-mm dishes coated with Gal8 for 0.5, 1, and 2 hr. Following incubation, cells were lysed, and protein extracts were subjected to electrophoresis in 12% SDS-PAGE gels. Blots were probed with anti-phosphorylated myosin light chain 2 (ppMLC2) (Thr18/Ser19) antibody. The blots were subsequently stripped and reprobed with anti-MLC antibody. A representative Western blot is shown in the top panel. Images were acquired by Odyssey Infrared Imaging System, and band intensity was quantified by ImageJ (bottom panel). N = 3. **B:** Phosphorylation of MLC2 is inhibited by Rho and ROCK inhibitors. Normal human TM cells were serum-starved overnight and treated with C3 transferase (2 µg/ml) and Y27632 (20 µM) for 4 hr. Treated cells were detached and plated on Gal8-coated dishes for 2 hr in the presence or absence of inhibitors and were then examined for the expression levels of ppMLC2 as described in the legend to panel A. Top: A representative Western blot; bottom: quantification of ppMLC2. Data are expressed as mean±SEM and analyzed with one-way ANOVA. ***P<0.001 vs control. N = 3.

A key regulator of actin cytoskeleton is the small GTPase Rho [24], [25], [26]. One of the effector proteins of Rho is the Rho-kinase, also known as Rho associated coiled coil kinase (ROCK). To assess the involvement of ROCK in the formation of stress fibers in TM cells adhered to Gal8, we conducted the cytoskeletal rearrangement assay in the presence of a well characterized ROCK inhibitor, Y27632. Y28632 significantly inhibited stress-fiber formation in a dose-dependent manner (Figure 4C). At 20 µM of Y27632, very few stress fibers formed in TM cells adhered to Gal8. Instead, almost every cell had protrusion of dendrite-like extensions (Figure 4D). At the highest concentration of the inhibitors used, C3 transferase and Y28632 inhibited the stress fiber formation by 50% and 95% respectively. The different effects of the two inhibitors may be due to the characteristics of the inhibitors. The ROCK inhibitor, Y27632, changes TM cell morphology within 2 hr. However, for the Rho inhibitor, C3 transferase, it takes at least 4 hr to changes TM cell morphology. We did not extend the incubation time beyond 2 hr because TM cells also produce other matrix proteins which may complicate interpretation of data. Despite these profound effects, neither Y27632 nor C3 transferase were cytotoxic to TM cells (Figure S1). Neither inhibitor had any effect on the actin cytoskeleton of cells adhered to poly-L-lysine (data not shown). Together, these results suggest that Gal8 promotes stress fiber formation in TM cells through activation of Rho signaling and that the pathway for this effect runs through ROCK. Activated ROCK promotes the accumulation of phosphorylated MLC2 which, in turn, promotes the formation of stress fibers [27]. To find whether MLC2 is phosphorylated in TM cells adhered to Gal8, we analyzed the phospho-MLC2 content of the cells with a MLC2 (Thr18/Ser19) antibody. TM cells adhered to Gal8 showed a time-dependent accumulation of phosphorylated MLC2 (Figure 5A). The extent of accumulating phosphorylated MLC2 on Gal8-coated wells was similar to that seen in cells adhered to fibronectin (Figure S2). Elevated level of phospho-MLC2 was detected at 1 hour and robust stress fibers were detected at 2 hours on Gal8 substrate. This is in line with the current understanding that stress fiber formation is downstream of, and, is modulated by phosphorylated MLC2. Treatment with Y27632 and C3 transferase abolished phosphorylation of MLC2 of TM cells on Gal8 substrate (Figure 5B). Taken together, these data suggest a novel activity for Gal8 involving induction of the Rho/ROCK/MLC2 pathway leading to cell spreading and stress fiber formation.

## Discussion

Perturbation of the outflow of the aqueous humor leads to elevation in intraocular pressure, which is one of the major causal risk factors for vision loss in POAG [1]. The dynamic structure of the TM cells actin cytoskeleton with its focal adhesions is an important determinant of outflow facility. Reagents that disrupt the actin cytoskeleton such as cytochalasins, lantrunculin and H-7, cause elevation in outflow facility as well as loss of TM cell contact with the beams and neighboring cells [28], [29], [30], [31]. Our data support the hypothesis that Gal8 can regulate TM actin cytoskeleton. TM cell adhesion to Gal8 was followed by cell spreading and actin cytoskeleton rearrangement, showing gradual actin polymerization into stress fibers over time in adhered cells. A key regulator of actin cytoskeleton is the small GTPase Rho [24], [25], [26]. Activated (GTP-bound) Rho interacts with ROCK and activates its kinase activity. This in turn, phosphorylates and inactivates MLC phosphatase, and also phosphorylates MLC directly. The phosphorylation of MLC leads to rearrangement of cytoskeleton and stress fiber formation [27]. Recent studies have shown that phosphorylation of MLC in TM cells is dependent on activation of the Rho/ROCK signaling pathway [10], [12]. In the current study, we demonstrate for the first time that Gal8 can modulate Rho GTPase signaling in TM cells. Our findings that spreading of TM cells on Gal8 is associated with the accumulation of phosphorylated MLC2 and that inhibitors of Rho as well as of ROCK attenuate TM cell spreading and stress fiber formation, suggest that Gal8 has the ability to participate in the regulation of the Rho/ROCK/MLC2 signaling pathway in TM cells. Rho signaling has been intimately linked to the regulation of outflow facility. Specific inhibitors, like ROCK, Y27632 [9], [11] and H1152 [10] have been shown to increase outflow facility in whole monkey and porcine eyes in a time- and dose-dependent manner. Likewise, in a perfused human eye, dominant negative RhoA [2] and dominant negative ROCK [4] caused a marked increase in outflow facility, TM cell rounding and detachment from the beams. In cultured TM cells, delivery of a dominant negative RhoA [2] and dominant negative ROCK [4] caused disruption of the actin cytoskeleton, TM cell rounding, loss of stress fibers and focal adhesions.

The current study has focused on characterization of Gal8 effects on normal TM cells. The relevance of these effects to the pathogenesis of POAG needs to be investigated further. The *LGALS8* gene encodes six different isoforms of Gal8, resulting from alternative splicing and the use of multiple polyadenylation signals [32]. Different isoforms differ in the length of their hinge peptide [32]. It has been shown that isoforms with a short hinge peptide are severely impaired in their biological activity, they are profoundly less capable of promoting cell adhesion and of inducing outside-in signaling [33]. In a recent study conducted in our lab [34], glycogene expression patterns were compared between normal and glaucomatous TM tissues, using a specialized glycogene microarray, GLYCOv2 (Consortium for Functional Glycomics). While this study revealed no differences in Gal8 expression levels between normal and glaucomatous TM tissues, the Gal8 probes used in the GLYCOv2 microarray were not designed to detect the alternatively spliced isoforms of Gal8 with the different hinge peptide lengths. Thus, one cannot eliminate the possibility that normal and glaucomatous TM may express distinct isoforms of Gal8 resulting in different effects on the tissues; longer-hinged Gal8 possess increased glycan-binding capacity, which may decrease TM cell relaxation and decrease aqueous outflow in POAG patients. In addition, a recent study has reported that non-synonymous single nucleotide polymorphism of Gal8 (F19Y) is strongly associated with autoimmune diseases, such as rheumatoid arthritis and myasthenia gravis [35]. In these autoimmune diseases, Gal8 expression level is not affected. Thus, a possibility that Gal8 polymorphism may be involved in the pathogenesis of glaucoma cannot be ignored. On the other hand, a different regulatory mechanism may involve not Gal8 itself but its countereceptors. TM cells treated with dexamethasone show elevated expression levels of α_5_ integrin [36] which we previously found to bind to Gal8 [17]. It is possible that glaucomatous TM cells bearing more countereceptors for Gal8 are more susceptible to its effects.

In conclusion, data presented in the current study suggest a novel function for Gal8 in activation of Rho signaling and the regulation of TM cell cytoskeleton. It lays a foundation for exploration of a possible role for Gal8 in regulation of aqueous outflow and the pathogenesis of POAG.

## Materials and Methods

### Immunohistochemical Detection of Galectin-8 in Human TM

Human eyes with no known history of eye disease were obtained from the National Disease Research Interchange (Philadelphia, PA) within 24 hr postmortem. The eyes were bisected by an equatorial incision and the anterior segment of the eye was cut into four quadrants. Tissues were fixed in formaldehyde (4%, 3 hr, 25°C) and were then processed for paraffin embedding. For immunostaining, longitudinal tissue sections (5 µm) were deparaffinized and sequentially treated with a basic pH antigen retrieval reagent (R&D systems, Minneapolis, MN), normal horse serum (R&D systems), goat anti-human Gal8 (2 hr, 37°C, R&D Systems; the goat antibody is specific for Gal8 and it does not recognize other galectins including Gal3 or Gal1), biotinylated anti-goat IgG (1 hr, 25°C, R&D Systems), a freshly prepared solution of avidin-biotin-complex (R&D Systems’ Cell and Tissue Staining Kit, 30 min, 25°C) and a diaminobenzidine/H_2_O_2_ reagent (R&D systems). Control sections were processed the same way except that either the step involving incubation with the primary antibody was omitted or primary antibody was substituted with nonimmune goat serum (R&D systems).

### Human TM Cell Cultures

Normal human cadaver eyes (donor age 71–89 years) were obtained from the Central Florida Lions Eye Bank (Tampa, FL). All eyes were enucleated within 6 hr postmortem and tissues were explanted for culture within 24 hr postmortem. TM cells were grown in tissue culture as described previously [37]. Cultures were propagated in DMEM supplemented with 10% FBS and cells were used at third to fourth passage. Consistent with published studies, these cells expressed aquaporin1 and CD44, and, when treated with Dexamethasone, markedly upregulated the expression of myocilin. (Data not shown, expression levels of CD44 and myocilin were detected by Western blot and RT-PCR analyses, respectively, and the expression of aquaporin1 was detected in a microarray study using 48.5 Illumina HEEBO oligo microarrays [Microarrays, Inc. Nashville, TN]). For some *in vitro* experiments, primary human TM cells purchased from ScienCell (Carlsbad, CA) were used.

### RT-PCR Amplification of Galectin-8

Confluent cultures of TM cells from two different donors were subjected to RNA extraction using the RNEasy RNA extraction kit (Qiagen, Valencia, CA). On-column DNase (Qiagen) digestion was performed during RNA purification to avoid any DNA contamination. Reverse transcription was performed on total RNA (1 µg) with random primers (Invitrogen) in the presence or the absence of reverse transcriptase (RT) (SuperScript II, Invitrogen). Primer sequences: 5′ ccc/tgt/tct/ctt/gag/ctt/cg 3′ and 5′ cac/tgg/gga/agg/agt/tgt/gt 3′, were chosen for an expected product size of 191 bp. PCR products were electrophoresed on 1% agarose gel, and sequenced at the Tufts University Core Facility.

### Quantitative RT-PCR

To assess the abundance of Gal8 expression in TM, the expression level of Gal8 was compared with that of GAPDH by quantitative RT-PCR. Quantitative RT-PCR was performed using the Mx4000 real-time PCR machine (Stratagene, La Jolla, CA). Briefly, cDNA was synthesized from 25 ng total RNA using the High Capacity cDNA Archive Kit (Applied Biosystems, Foster City, CA) according to the manufacturer’s instructions. PCR was carried out in triplicates using inventory gene-specific primers (Gal8:HS00180706; GAPDH: HS99999905, Applied Biosystems) and the TaqMan Universal PCR Master Mix containing ROX as a passive reference. Reactions performed in the absence of template served as negative controls. Fluorescent signals were recorded once per cycle with a detector corresponding to FAM. To normalize the non-PCR related fluctuations between wells, each fluorescent reporter signal was measured against the ROX (internal reference dye) signal. Amplification plots showing the increase in the FAM fluorescence with each cycle of PCR (ΔRn) were generated and threshold cycle values (Ct) were calculated for all samples. The Ct value represented the PCR cycle number at which the fluorescence was detectable above a threshold based on the variability of the baseline data during the first 15 cycles. All Ct values were obtained in the exponential phase.

### Western Blot Analysis of Galectin-8

To detect the expression of Gal8 in TM cells, 0.6 ml of cold radioimmunoprecipitation (RIPA) buffer containing a protease inhibitor cocktail (Roche Applied Science, Mannheim, Germany) was added to washed confluent cell cultures. Since Gal8 is a carbohydrate binding protein with high affinity towards β-lactose, a β-lactose affinity chromatography assay was used. After 30 min on ice, cell extracts were clarified by centrifugation and were incubated with Sepharose beads conjugated with β-lactose (1 hr, 4°C; EY labs, San Mateo, CA). Following the incubation period, the beads were washed with PBS containing 0.1% Triton X-100 (PBS-T), eluted first with a non-competing sugar, sucrose (100 mM in PBS-T) towards which Gal8 has no affinity, and then with β-lactose (100 mM in PBS-T). Eluates were dialyzed and then analysed by SDS-PAGE. Protein blots were stained with Ponceau S (Sigma) and were then processed for immunostaining using goat anti-human Gal8 as a primary antibody (1 hr, 25°C; R&D Systems), peroxidase-labeled anti-goat IgG (Vector Labs) as a secondary antibody, and a chemiluminescence detection system (Perkin Elmer, Boston, MA).

### Preparation of Recombinant Human Glutathione-S-Transferase Tagged Galectin-8

Recombinant human glutathione-S-transferase (GST) tagged Gal8, was produced and purified as previously described [14] with a few modifications. Lysates of bacteria expressing GST-Gal8 (2L culture) were chromatographed on a β-lactose-conjugated Sepharose column (EY Labs; 1 ml bed volume). After allowing Gal8 to bind to the affinity matrix, the gel bed was washed first with wash buffer I (20 ml of 20 mM Tris-HCl pH 7.4, 150 mM NaCl, 2 mM EDTA, 0.2 mM PMSF, 4 mM β-mercaptoethanol) and then with 10 ml of wash buffer II (PBS containing 0.2 mM PMSF, 4 mM β-mercaptoethanol). GST-Gal8 was eluted from the column with 10 ml wash buffer II containing 100 mM β-lactose. Fractions containing the lectin were dialyzed against PBS containing 2% glycerol and 4 mM β-mercaptoethanol and stored at −80°C.

### Quantification of Cell Adhesion

To assess the adhesion and spreading of TM cells onto different substrates, 96-well microtiter plates were coated with Gal8 (20 µg/ml), human fibronectin from placenta (Sigma) (20 µg/ml), and poly-L-lysine (Sigma) (100 µg/ml). After 2 hr, plates were blocked with 1% BSA/Dulbecco’s PBS (DPBS) (1 hr, 25°C). Confluent TM cultures were dissociated with Accutase (Invitrogen), resuspended in 0.2% BSA/DPBS, and plated on microtiter plates coated with Gal8 and other substrates described above (30,000 cells/well, 3 wells/group). Following incubation at 37°C for 30 min, cells were washed with PBS, stained/fixed with 0.2% crystal violet (Sigma) in PBS containing 20% methanol (15 min, 25°C), and washed thoroughly with distilled water to remove unbound stain. Bound stain was solubilized with 1% SDS in distilled water for 1 hr, and the absorbance was measured at 595 nm by the FilterMax F5 Multi-Mode Microplate Reader (Molecular Devices, Sunnyvale, CA). To assess the involvement of β_1_-integrins in the adhesion of TM cells on Gal8, cells were incubated in the presence of an anti-integrin β_1_ functional-blocking antibody, JB1A (1∶50 dilution; 10 min, 25°C; EMD Millipore, Billerica, MA). The JB1A antibody binds to a specific regulatory epitope (amino acids: 82 to 87) on β1 integrin that is distinct from the RGD binding site (amino acids: 140–164) [38]. The antibody binding to this epitope locks the integrin in an inactive conformation and does not allow it to be activated regardless of the nature of the ligand presented [39]. This antibody has been shown to interfere with the adhesion of various cell types to a number of ECM substrates including fibronectin and collagen I [18], [19], [40], [41], [42].

### Quantification of Cell Spreading and F-actin Staining

Human TM cells plated on Gal8 substrates were incubated in the presence of JB1A antibody for 30 min as described in the previous section. At the end of incubation period, cells were fixed with 4% paraformaldehyde in PBS (15 min, 25°C), washed with PBS three times, and permeabilized with 0.5% Triton X-100 in PBS (5 min, 25°C). After washing again with PBS three times, non-specific binding sites were blocked with 1% BSA in PBS (20 min, 25°C), the cells were stained with rhodamine-labeled phalloidin (100 nM in PBS; Cytoskeleton, Denvor, CO), mounted in Vectashield mounting medium with DAPI (Vector Laboratories, Burlingame, CA). Six to eight different fields of each experimental condition were photographed, and the spread areas of individual cells were quantified by ImageJ software (National Institute of Health, Bethesda, MD).

### Detection of Cytoskeletal Changes in Human TM Cells Adhered to Galectin-8

Eight-chamber glass slides were coated with Gal8 (20 µg/ml), human fibronectin from placenta (Sigma) (20 µg/ml), and poly-L-lysine (Sigma) (100 µg/ml). TM cultures were dissociated with Accutase and plated on substrate-coated slides (5000 cells/chamber in serum-free DMEM). Following incubation at 37°C for 0.5, 1, or 2 hr, cells were washed, fixed and stained with rhodamine-labeled phalloidin as previously described. To assess the involvement of Rho signaling in stress fiber formation in cells adhered to Gal8, TM cells were serum starved overnight and treated with different concentrations of ROCK inhibitor, Y27632 (Abcam, Cambridge, MA) or the Rho-specific inhibitor, C3 transferase from clostridium botulinum (Cytoskeleton) for 4 hr. This enzyme is a highly specific inhibitor of Rho that does not affect other Rho family members such as Rac or cdc42. It is a ribosyltransferase that by modifying the Asn 41 residue of Rho, renders it biologically inactive [43].

### Detection of Myosin Light Chain Phosphorylation in TM Cells Adhered to Galectin-8

In an effort to further confirm the involvement of Rho signaling in stress fiber formation in TM cells adhered to Gal8, confluent TM cell cultures were dissociated with Accutase and plated on Gal8-coated dishes (100 mm). After incubation at 37°C for 0.5, 1 and 2 hr, cells were lysed with cell lysis buffer (Cell Signaling Technology, Danvers, MA) supplemented with the complete protease inhibitor cocktail (Roche Applied Science) and PhosSTOP phosphatase inhibitor cocktail (Roche), and subjected to electrophoresis in 12% SDS-PAGE gels. Protein blots of the gels were blocked with Odyssey® blocking buffer (OBB) (Li-COR, Biosceiences, Lincoln, NE) and were then probed with rabbit anti-phospho-myosin light chain 2 (Thr18/Ser19) primary antibody (1∶500 dilution in OBB, 4°C, overnight; Cell Signaling Technology), followed by anti-rabbit IRDye 800CW secondary antibody (Li-COR) (1∶10,000 dilution in OBB, 45 min, room temperature). Membranes were scanned by an Odyssey® Infrared Imaging System using Image Studio v2.0 software (Li-COR). After image acquisition, membranes were stripped with the NewBlot nitrocellulose stripping buffer (Li-Cor) and re-probed with rabbit anti-MRCL3/MRLC2/MYL9 (FL-172) primary antibody (Santa Cruz Biotechnology, Santa Cruz, CA) (1∶400 dilution) followed by anti-rabbit IRDye 800CW secondary antibody. Relative band intensity was quantified by ImageJ.

## Supporting Information

Figure S1 **Rho and ROCK inhibitors are not cytotoxic to TM cells.** **A:** Cells were incubated on glass slides coated with recombinant human Gal8 in the absence (a) or the presence of: Y27632 at 20 µM (b), or C3 transferase at 2 µg/ml (c) or tert-butyl hydroperoxide (tBH) at 3.5 mM (d). Following the incubation period, cells were washed and stained with ethidium homodimer III (red) which stains dead cells and Hoechst 33342 (blue) which stains nuclei of both living and dead cells. In the left panel are representative micrographs from each group showing no significant cell death in the presence of Y27632 (b) or (c) C3 transferase and significant cell death in the presence of tBH (d). Random fields of each experimental condition were photographed, and dead cells were counted manually. Percent dead cells for cells adhered to Gal8 in the presence of the different inhibitors are shown in panel B. Data are shown as mean ± SEM and analyzed by one-way ANOVA. N = 3.
(TIF)

Figure S2 **Galectin-8 promotes phosphorylation of myosin light chain.** Normal human TM cells were incubated on Gal8-coated 100-mm dishes for 0.5, 1, 2 and 4 hr. Following incubation, cells were lysed, and protein extracts were subjected to affinity chromatography using a phosphoprotein affinity column. Bound fraction was electrophoresed on SDS-polyacrylamide gel and gel blots were stained with Ponceau S (A) and were then processed for immunostaining with anti-myosin light chain antibody (B). Phosphoproteins isolated from cells incubated on fibronectin and poly-L-lysine for 4 hr served as positive and negative controls respectively. The expected MLC band of 20-kDa appeared in the phosphorylated fraction of all cell lysates. Approximate band intensity was quantified by image analysis software and normalized to Ponceau S staining. The accumulation of phosphorylated MLC over time in TM cells adhered to Gal8 is plotted in panel C. Comparison of phosphorylated MLC content in cells adhered to different substrates following 4 hr incubation is shown in panel D. Note that by 4 hr, MLC phosphorylation is similar in cells adhered to Gal8 and to fibronectin. Lys, poly-L-lysine; FN, human fibronectin.
(TIF)
