# Supplementary figures and images for: Galectin-8 Promotes Cytoskeletal Rearrangement in Trabecular Meshwork Cells through Activation of Rho Signaling
Source: PLoS One. 2012 Sep 4;7(9):e44400. doi: 10.1371/journal.pone.0044400 (PMC3433423; doi:10.1371/journal.pone.0044400)

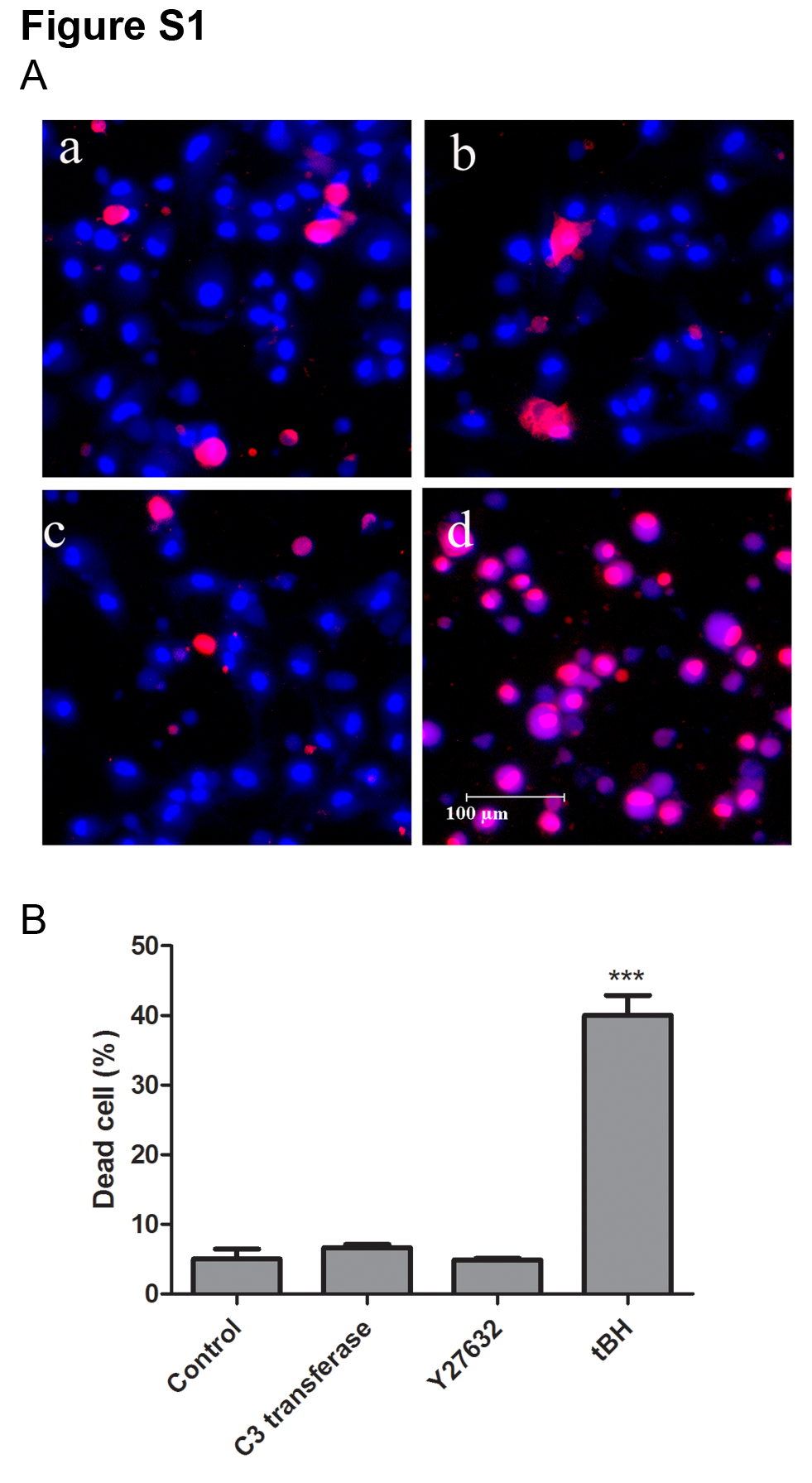

Supplement: Figure S1 — Rho and ROCK inhibitors are not cytotoxic to TM cells. A: Cells were incubated on glass slides coated with recombinant human Gal8 in the absence (a) or the presence of: Y27632 at 20 µM (b), or C3 transferase at 2 µg/ml (c) or tert-butyl hydroperoxide (tBH) at 3.5 mM (d). Following the incubation period, cells were washed and stained with ethidium homodimer III (red) which stains dead cells and Hoechst 33342 (blue) which stains nuclei of both living and dead cells. In the left panel are representative micrographs from each group showing no significant cell death in the presence of Y27632 (b) or (c) C3 transferase and significant cell death in the presence of tBH (d). Random fields of each experimental condition were photographed, and dead cells were counted manually. Percent dead cells for cells adhered to Gal8 in the presence of the different inhibitors are shown in panel B. Data are shown as mean ± SEM and analyzed by one-way ANOVA. N = 3. (TIF) [file pone.0044400.s001.tif]

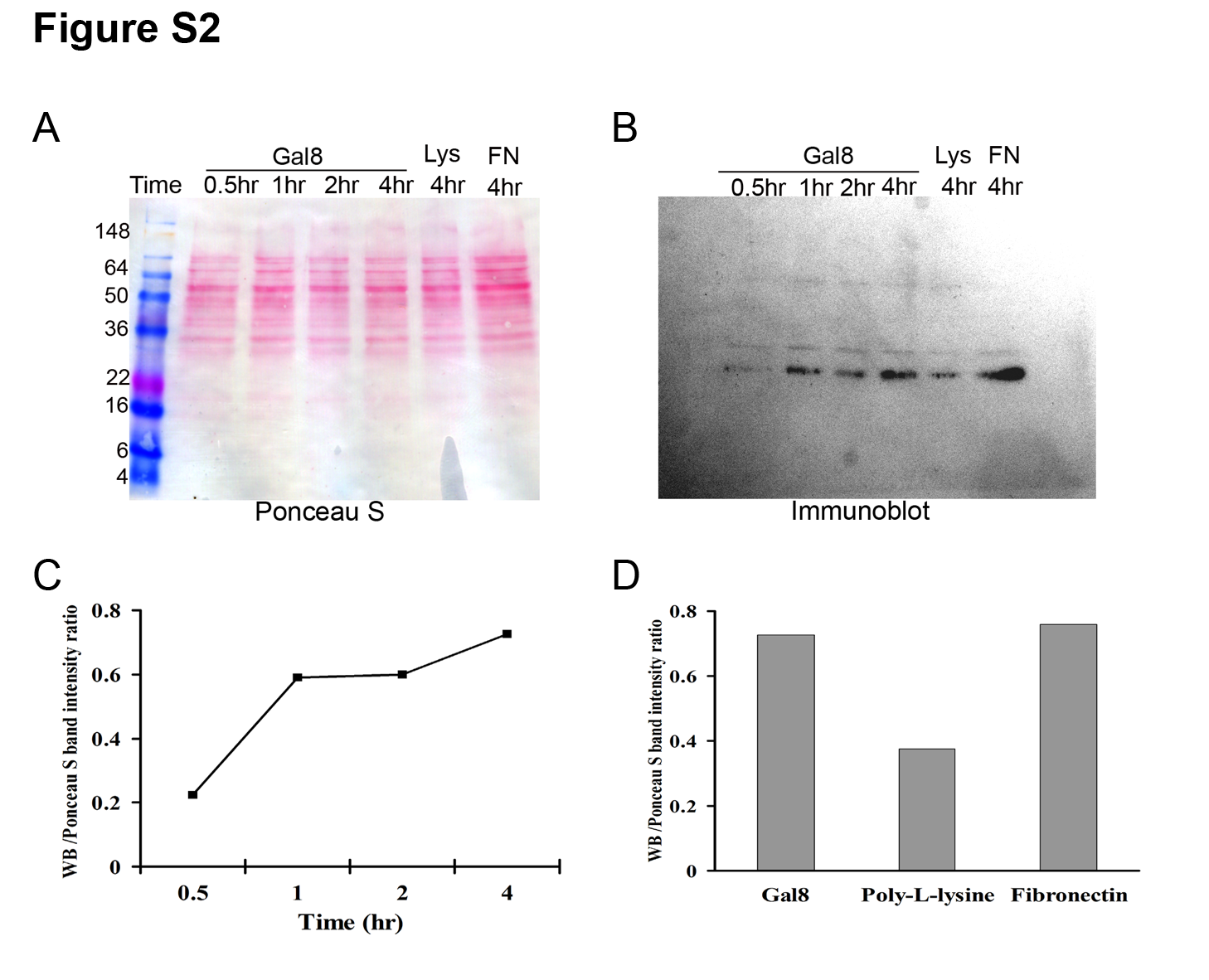

Supplement: Figure S2 — Galectin-8 promotes phosphorylation of myosin light chain. Normal human TM cells were incubated on Gal8-coated 100-mm dishes for 0.5, 1, 2 and 4 hr. Following incubation, cells were lysed, and protein extracts were subjected to affinity chromatography using a phosphoprotein affinity column. Bound fraction was electrophoresed on SDS-polyacrylamide gel and gel blots were stained with Ponceau S (A) and were then processed for immunostaining with anti-myosin light chain antibody (B). Phosphoproteins isolated from cells incubated on fibronectin and poly-L-lysine for 4 hr served as positive and negative controls respectively. The expected MLC band of 20-kDa appeared in the phosphorylated fraction of all cell lysates. Approximate band intensity was quantified by image analysis software and normalized to Ponceau S staining. The accumulation of phosphorylated MLC over time in TM cells adhered to Gal8 is plotted in panel C. Comparison of phosphorylated MLC content in cells adhered to different substrates following 4 hr incubation is shown in panel D. Note that by 4 hr, MLC phosphorylation is similar in cells adhered to Gal8 and to fibronectin. Lys, poly-L-lysine; FN, human fibronectin. (TIF) [file pone.0044400.s002.tif]
